# Deactivation vs. asynchronous pacing - prospective evaluation of a protocol for rhythm management in patients with magnetic resonance conditional pacemakers undergoing adenosine stress cardiovascular magnetic resonance imaging

**DOI:** 10.1186/s12872-017-0579-1

**Published:** 2017-06-02

**Authors:** Oliver Klein-Wiele, Marietta Garmer, Gianluca Barbone, Rhyan Urbien, Martin Busch, Kaffer Kara, Harald Schäfer, Michael Schulte-Hermes, Birgit Hailer, Dietrich Grönemeyer

**Affiliations:** 1 Department of Cardiology, Katholisches Klinikum Essen, University of Witten/Herdecke, Hülsmannstraße 17, 45355 Essen, Germany; 2 Department of Radiology, Grönemeyer Institut Bochum, University of Witten/Herdecke, Universitätsstraße 142, 44799 Bochum, Germany; 3 Cardiovascular Center, Josef Hospital, University of Bochum, Gudrunstr. 56, 44791 Bochum, Germany; 4 Department of Cardiology, Prosper-Hospital Recklinghausen, University of Witten/Herdecke, Mühlenstraße 27, 45659 Recklinghausen, Germany

**Keywords:** Pacemaker, MRI conditional, Cardiovascular magnetic resonance, CMR, Adenosine stress, Safety, Sinus node dysfunction, Atrioventricular block, Asynchronous pacing

## Abstract

**Background:**

Cardiovascular Magnetic Resonance (CMR) imaging with adenosine stress is an important diagnostic tool in patients with known or suspected coronary artery disease (CAD). However, the method is not yet established for CAD patients with pacemakers (PM) in clinical practice. A possible reason is that no recommendations exist for PM setting (paused pacing or asynchronous mode) during adenosine stress. We elaborated a protocol for rhythm management in clinical routine for PM patients that considers heart rate changes under adenosine using a test infusion of adenosine in selected patients.

**Methods:**

47 consecutive patients (mean age 72.3 ± 10,0 years) with MR conditional PM and known or suspected CAD who underwent CMR in clinical routine were studied in this prospective observational study. PM indications were sinus node dysfunction (SND, *n* = 19; 40,4%), atrioventricular (AV) block (*n* = 26; 55.3%) and bradyarrhythmia in permanent atrial fibrillation (AF, *n* = 2; 4.3%). In patients with SND, normal AV-conduction and resting HR >45 bpm at the time of CMR and in AF the PM was deactivated for the scan. In intermittent AV-block a test infusion of adenosine was given prior to the scan. All patients with permanent higher degree sinuatrial or AV-block or deterioration of AV-conduction in the adenosine test were paced asynchronously during CMR, in patients with preserved AV-conduction under adenosine the pacemaker was deactivated. CMR protocol included cine imaging, adenosine stress perfusion and late gadolinium enhancement.

**Results:**

The adenosine test was able to differentiate between mandatory PM stimulation during CMR and safe deactivation of the device. In patients with permanent sinuatrial or AV-block (*n* = 11; 23.4%) or deterioration of AV conduction in the adenosine test (*n* = 5, 10.6%) asynchronous pacing above resting heart rate did not interfere with intrinsic rhythm, no competitive stimulation was seen during the scan. 10 of 15 (66,7%) patients with intermittent AV-block showed preserved AV-conduction under adenosine. As in SND and AF deactivation of the PM showed to be safe during CMR, no bradycardia was observed.

**Conclusion:**

Our protocol for rhythm management during adenosine stress CMR showed to be feasible and safe and may be recommended for pacemaker patients undergoing routine CMR.

## Background

CMR as a non-invasive imaging modality is firmly established in the clinical workup for patients with known or suspected CAD. Beside cine imaging and scar detection with Late Gadolinium Enhancement (LGE) ischemia detection plays a major role in CAD [1, 2]. It has a class Ia level A recommendation in case of intermediate pre test probability of CAD in latest guidelines [2]. Hemodynamic relevance of stenoses in known CAD can be evaluated reliably [3].

In patients with cardiac conduction disorders underlying or concomitant CAD is common [4, 5]. However, while safety of CMR without stress agents in patients with MR conditional PM has been shown in a number of studies [6–8] no prospective data exists on rhythm management of those patients in adenosine stress CMR.

In MR conditional PM no inhibited mode can be chosen. Only deactivation (ODO-mode) or asynchronous pacing (DOO, AOO, VOO) [9] are available to avoid inhibition by electromagnetic interference or tracking of electromagnetic impulses. In SND and AV-block selecting an adequate pacing mode for routine adenosine stress CMR can be challenging because the effect of adenosine on heart rate (HR) has not been thoroughly studied in this entity of patients. Asynchronous mode (i.e. pacing at a fixed rate above baseline HR) could result in competitive pacing when HR accelerates under adenosine [10] reaching the pacing rate. This is due to the fact that sensing is deactivated in this mode and intrinsic rhythm cannot inhibit PM activity. PM stimuli falling in the vulnerable period of the cardiac cycle could then trigger arrhythmia [11]. On the other hand deactivation of the PM in patients with normal HR under resting conditions could result in bradycardia or asystole under adenosine administration [12].

We recently published first data on adenosine stress CMR in pacemaker patients in a small retrospective study and found no relevant complications under a predefined pacing protocol [13]. However, the number of patients with AV-block was limited and it remains unclear what pacing mode is preferable for patients with intermittent AV-block i.e. without higher degree AV-block at the time of CMR. To further investigate adenosine response and to decide whether the pacemaker can be deactivated during the scan we used a test infusion of adenosine for patients with intermittent AV-block and preserved AV-conduction at the time of CMR to screen for adenosine induced bradycardia.

In conclusion the diagnostic value of perfusion analysis by CMR in CAD is well known but still no recommendations exist for PM programming in MRI conditional devices during adenosine stress. We sought to test the safety of a protocol for rhythm management in those patients that is adapted to the adenosine response in PM patients with different underlying conduction disorders.

## Methods

In this prospective observational study we analyzed 47 consecutive patients with MR conditional PM who underwent routine adenosine stress CMR including cine imaging, adenosine stress perfusion and LGE from april 2015 to december 2016. Inclusion criteria were presence of a MR conditional pacemaker system, indication for adenosine stress CMR for the evaluation of known or suspected CAD, age > 18 years. Exclusion criteria were implantation <6 weeks from the scan, epicardial or abandoned pacemaker leads, bronchial, asthma, impaired renal function, ejection fraction <35%, presence of an antitachycardia device.

### General characteristics

Patients had a mean age of 72.1 ± 11.0 years. 19 (40.4%) had known CAD, 8 (17.0%) previous MI. All other patients had intermediate pretest probability of CAD [14]. Echocardiography had shown preserved systolic left ventricular (LV) function in all subjects. Pacemaker indications were sinus node dysfunction (SND, 19; 40.4%), second or third degree AV-block (*n* = 26; 55.3%) and bradyarrhythmia in permanent AF (*n* = 2; 4.3%). Eight patients (17.0%) were PM dependent (HR <30 bpm). Impulse generator/lead models were Advisa (*n* = 1; 2.1%) and Ensura (*n* = 46; 97.9%) MRI SureScan/CapSureFix 5076 Novus (atrial), CapSureSense 4074 (ventricular) (Medtronic Inc., Minneapolis, MA, USA). For detailed baseline characteristics see Table 1.

**Table 1:** Baseline characteristics

Total patients	47	
Mean Age (years)	72.3 ± 10.0	
	N	%
Female	22	46.8
Pacemaker indication		
Higher degree AV Block	26	55.3
Sinus node dysfunction	19	40.4
Bradyarrhythmia in AF	2	4.3
Coronary artery disease	19	40.4
Previous MI	8	17.0
Paroxysmal atrial fibrillation	17	36.2
In AF at the time of CMR	2	4.3
Hypertension	40	85.1
Impaired renal function	2	4.3
Previous Stroke	7	15.0
Pacemaker		
Ensura MRI Sure Scan	46	97.9
Advisa DR MRI Sure Scan	1	2.1
Pacemaker dependent	8	17.0

AV, atrioventricular, AF, atrial fibrillation, MI, myocardial infarction, CMR, cardiovascular magnetic resonance

### Pacemaker programming and adenosine test

CMR was performed more than six weeks after PM implantation in all individuals according to ESC guidelines [15]. Prior to and after CMR imaging battery status of the device, lead impedance, pacing capture thresholds and sensing amplitudes were measured. In patients with intermittent AV-block and preserved AV-conduction at the time of CMR PQ-interval was measured in resting ECG and the Wenckebach point was assessed by testing in AOO mode via the PM programmer. A Wenckebach point below 120 bpm was considered pathologic. Furthermore these patients received a test infusion of adenosine as 3-min infusion of 140μg/kg body weight/min according to the dose for CMR. The pacemaker was deactivated for the test (ODO). Possible deterioration of AV-conduction was recorded and classified in progression to second degree AV-block II Mobitz I, second degree AV-block Mobitz II and third degree AV-block. Reactivation of pacing was performed immediately in case of bradycardia. In SND the PQ interval and the Wenckebach point were measured to rule out occult AV-nodal disease; as in AF no adenosine test was performed prior to MR.

Devices were programmed to MR safe mode following manufacturer’s instructions immediately prior to the scan and reprogrammed immediately thereafter. Programming was performed according to a predefined protocol: To avoid interference of intrinsic rhythm with PM-stimulation in patients with SND and resting heart rate HR > 45 bpm no pacing (ODO)-mode was engaged during the scan - also when atrial fibrillation (AF) was present. In individuals with SND and HR ≤ 45 bpm the pacemaker was set to asynchronous atrial stimulation (AOO, 60 bpm). All patients with permanent second or third degree AV block and those with deterioration of AV-conduction in the adenosine test were continuously paced in asynchronous mode. Pacing rate was set 10 bpm above spontaneous heart rate with a minimum of 60 bpm. VOO mode at 60 bpm was chosen in AV block with sinus rate > 45 bpm to avoid competitive atrial stimulation, DOO mode at 60 bpm in AV block with sinus bradycardia ≤45 bpm. In patients with intermittent AV-block and preserved AV-conduction (i.e. no progression to second or third degree AV-block in the adenosine test) the PM was deactivated as in SND. Patients in AF at the time of the scan were paced VOO at 60 bpm if resting heart rate was ≤45 bpm. Table 2 shows the protocol used to select pacing modes for specific clinical constellations.

**Table 2:**
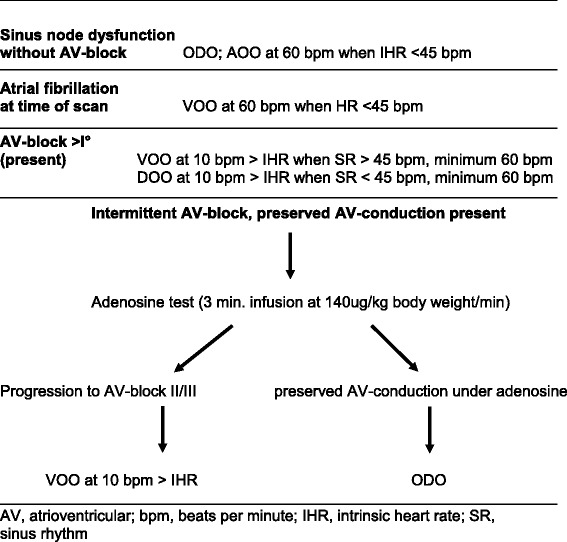
Protocol for the selection of pacing modes

### Safety precautions

As previously described [13] patients were monitored during the scan with continuous electrocardiographic and visual supervision by a cardiologist present in the scanner room. Voice contact was maintained with the patient at all times of the scan. Advanced cardiac life support protocol was in effect. In the scanner the patient was placed on a carry sheet; medical staff was trained for rapid removal of the patient from the scanner room in the event of cardiopulmonary compromise. Thus immediate treatment of severe arrhythmia and reactivation of PM stimulation within seconds in non-paced patients was guaranteed. Atropine, adrenaline and theophylline injections were prepared ready for use in case of bradycardia. Two separate cubital venous cannulas were used for adenosine and gadolinium contrast agent respectively.

### Cardiac magnetic resonance imaging

CMR scans were performed with a 1.5 T wide bore system (ESPREE – Siemens Healthcare, Erlangen, Germany) using a 4-channel body array and an 8-channel spine coil. Maximum gradient field was 33 mT/m (Z-Engine) with a slew rate of 100 T/m/*S. maximum* specific absorption rates were limited to 2.0 W/kg.

As previously published [13] our standard protocol meets the Society of Cardiovascular Magnetic Resonance (SCMR) guidelines for CMR [16]. Cine steady-state free precession (SSFP) gradient-echo images were obtained in 10 to 12 short axis slices depending on the size of the ventricles and in 3 long axis planes corresponding to two, three and four chamber views. For stress perfusion-imaging adenosine was administered as 3-min infusion of 140μg/kg body weight/min. First-pass perfusion imaging was carried out with intravenous bolus administration of gadolinium (0.2 mmol/kg body weight) in a fast low angle shot (FLASH) sequence (3 to 4 slices). Late Gadolinium Enhancement (LGE) images were acquired fifteen minutes after injection of gadolinium as phase-sensitive inversion-recovery (PSIR) in short (10 to 12 slices) and long axis (3 planes) views.

## Results

### Adenosine test prior to CMR

**Fig. 1:**
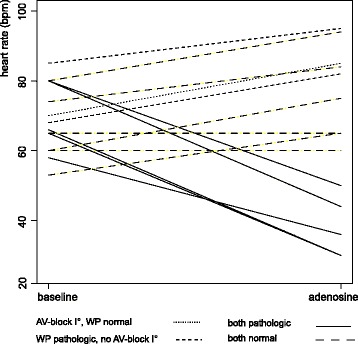
Adenosine test in intermittent AV-block. Individual changes of heart rate in patients with intermittent AV-block and currently preserved AV-conduction under a 3 min infusion of adenosine at 140μg/kg body weight/min. PQ-interval and Wenckebach point was assessed before the test and is indicated by different lines. Progression to AV-block II/III was only observed in patients with both prolonged PQ-interval (AV-block I°) and pathologic Wenckebach point (< 120 bpm). AV, atrioventricular; WP, Wenckebach point; bpm, beats per minute

15 patients (31.9%) with intermittent AV-block and preserved AV-conduction at the time of CMR were tested. 5 of them (33.3%) showed deterioration of AV conduction with a drop of HR. All of those individuals had both prolonged PQ-interval (220 to 305 ms) and pathologic Wenckebach point (70 to 90 bpm). Two patients showed worsening of AV-conduction to AV-block II Mobitz II, three patients developed AV-block III. One patient had pathologic PQ-interval (230 ms) and normal Wenckebach point (130 bpm) and showed acceleration of HR from 70 to 85 bpm without progressive AV-block. One patient had normal AV-conduction (190 ms) and pathologic Wenckebach point (115 ms) and showed an increase in HR from 85 to 95 bpm. Seven of 15 patients with intermittent AV-block (46.6%%) had normal PQ-interval and normal Wenckebach point. None of them showed higher degree AV-block in the test, heart rate increased from 66 ± 8.6 to 75 ± 11.4 bpm (*p* < 0.001, paired t-test). Thus only in patients with both pathologic PQ-interval and pathologic Wenckebach point worsening of AV-conduction and drop of HR were observed. The adenosine test was well tolerated by the patients; no adverse reactions were seen. Figure 1 summarizes heart rate response under the test infusion of adenosine.

### Effect and safety of adenosine administration for stress perfusion

There were no adenosine induced adverse events. Asynchronous stimulation of patients with permanent AV-block or progressive AV-block under adenosine 10 bpm above resting HR did not cause competitive pacing or arrhythmia during MR. Patients with preserved AV-conduction under adenosine and deactivated PM did not develop higher degree AV-block in CMR, HR increased from 67.6 ± 9.1 to 77.6 ± 11.3 bpm. Two patients with SND were in AF at the time of CMR. In patients with SND and normal AV-conduction i.e. normal PQ interval and normal Wenckebach point (*n* = 17; 36.2%) at the time of the scan with deactivated (ODO) pacemaker adenosine administration accelerated HR from 60.1 ± 9.1 to 76.0 ± 9.3 bpm (*p* < 0.001, Wilcoxon signed rank test). AV-conduction was not significantly influenced by adenosine; no higher degree AV block occurred. When sinus rate was <45 bpm (*n* = 1; 2.1%) AOO pacing at 60 bpm led to permanent capture, no acceleration of HR under adenosine was noticed. Of 4 patients in AF in 3 HR remained unchanged during stress perfusion, one patient had acceleration of HR from 110 to 125 bpm.

### Device integrity

Device integrity was not compromised by the CMR scan. No patient showed relevant alterations of lead impedance, pacing capture threshold, sensing amplitude or battery voltage. Table 3 summarizes device parameters pre and post CMR.

**Table 3:** Comparison of device parameters before and after CMR

	before MR	after MR	*P* ^a^
P-wave amplitude (mV)	2.87 ± 1.86	3.10 ± 1.70	0.32
R-wave amplitude (mV)	12.27 ± 5.32	12.05 ± 5.44	0.59
Atrial lead impedance (Ohm)	469 ± 61	468 ± 65	0.65
Ventricular lead impedance (Ohm)	601 ± 120	603 ± 118	0.57
Atrial PCT (V@0.4 ms)	0.66 ± 0.25	0.66 ± 0.20	1.0
Ventricular PCT (V@0.4 ms)	0.63 ± 0.26	0.55 ± 0.28	0.1
Battery voltage (V)	2.97 ± 0.42	2.97 ± 0.42	n.a.

CMR, Cardiovascular Magnetic Resonance; PCT, pacing capture threshold, ^a^Wilcoxon signed rank test

## Discussion

Adenosine stress CMR in patients with MR conditional PM is complicated by the absence of an inhibited pacing mode in these devices. Choosing an adequate PM setting (deactivation vs. asynchronous pacing) in specific conduction disorders can be challenging due to possible alterations of HR and heart rhythm under adenosine. Until now no guidelines for clinical routine have been established addressing this issue. We therefore elaborated a protocol for rhythm management intending to minimize the risk of arrhythmia due to competitive PM stimulation on the one hand and possible bradycardia on the other. In this study our protocol proved to be safe and feasible. No complications of adenosine stress CMR related to the presence of a PM or the underlying cardiac conduction disorder occurred.

The present study is in line with our previous findings [13] that in SND with normal resting HR and normal PQ interval paused PM stimulation (ODO mode) is suitable for adenosine stress perfusion. We found predominance of the sympatho-excitatory effect of adenosine [17] that overrides cardiac inhibition comparable to patients without SND resulting in a significant increase in HR. We selected DOO pacing only in patients with sinusbradycardia <45 bpm to prevent competitive atrial stimulation which can induce AF [18], also considering the fact that adenosine may promote AF by shortening the atrial action potential and refractory period [19]. Avoiding competitive atrial stimulation by pacing above intrinsic HR is not useful due to the acceleration of HR under adenosine (up to 40 bpm in tis study). Pacing far above baseline HR for a longer time could cause discomfort or even circulatory compromise in PM patients adapted to relative bradycardia [20]. VOO mode was avoided because ventricular tachycardia can be induced by R-on-T PM stimulation [21]. The risk of proarrhythmia with asynchronous ventricular pacing for PM interrogation is considered low [22]. However, routine adenosine stress CMR in CAD requires a long period in MR conditional mode because sequences for localization, cine imaging, first pass perfusion and LGE are necessary. Asynchronous ventricular PM stimulation might be hazardous under these conditions. In patients with higher degree AV-block or bradycardia induced by adenosine in the preceding test the risk of R-on-T stimulation considered low because the patient is paced above intrinsic HR at rest and under adenosine. Prolonged asystole under adenosine stress imaging in occult SND has been reported [23], therefore immediate reactivation of the PM in case of persistent bradycardia must be guaranteed. For further clinical practice we recommend deactivation of the device under careful monitoring in SND without AV-block.

The adenosine test in patients with intermittent AV-block was useful to differentiate patients that can be examined with deactivated devices from those with mandatory PM stimulation. One could argue that asynchronous pacing for all patients with AV-block may be chosen. However, with intact AV-conduction under adenosine the risk of proarrhythmia due to competitive ventricular stimulation cannot be excluded. Deterioration of AV-conduction was only observed in patients with both prolonged PQ interval and pathologic Wenckebach point. Larger studies have to show if CMR can be performed without previous testing in patients without first degree AV-block at rest and normal Wenckebach point. We believe that screening for occult AV-dysfunction by measuring the PQ-interval and Wenckebach point prior to CMR in SND is useful because SND patients may develop binodal disease [24] that does not become clinically apparent due to the PM therapy. We recommend adenosine test for SND patients with pathologic AV-conduction at the time of CMR, however all individuals in this study had normal AV-conduction. Further studies have to show if adenosine testing is adequate for patients with SND and impaired AV-conduction unmasked prior to CMR.

The investigated pacing protocol showed to be feasible and safe. However, adenosine stress CMR has to be compared to other diagnostic strategies, namely when the high supervisory expense in this setting is considered. We encourage prospective randomized studies to clarify which imaging strategy is the best choice for PM patients in terms of safety and clinical value.

### Limitations

This study is limited by the relatively sample size. Adverse effects may only appear in a larger cohort of patients. Furthermore we did not test a high dose adenosine protocol, which may be more effective in ischemia detection [25].

## Conclusion

Our protocol for rhythm management in patients with MR conditional PM appears to be feasible and safe and may be used for adenosine CMR in clinical routine until larger studies exist.

## Abbreviations

AV: Atrioventricular
BV: Battery voltage
CAD: Coronary artery disease
CMR: Cardiovascular Magnetic Resonance
ESC: European Society of Cardiology
FLASH: Fast low angle shot
HR: Heart rate
LGE: Late Gadolinium Enhancement
MI: Myocardial infarction
PCT: Pacing capture threshold
PM: Pacemaker
PSIR: Phase-sensitive inversion recovery
SCMR: Society for Cardiovascular Magnetic Resonance
SND: Sinus node dysfunction
SSFP: Steady-state free precession
STIR: Short-tau inversion recovery
